# Effect of caesarean section on maternal and foetal outcomes in acute fatty liver of pregnancy: a systematic review and meta-analysis

**DOI:** 10.1038/srep28826

**Published:** 2016-07-08

**Authors:** Hong-Yan Wang, Qing Jiang, Hao Shi, Yun-Qing Xu, Ai-Chao Shi, Yuan-Li Sun, Jian Li, Qin Ning, Guan-Xin Shen

**Affiliations:** 1 Department of Infectious Disease, Tongji Hospital of Tongji Medical College, Huazhong University of Science and Technology, Wuhan, China; 2 Department of Immunology, School of Basic Medicine, Tongji Medical College, Huazhong University of Science and Technology, Wuhan, China; 3 Department of Epidemiology and Biostatistics, and the Ministry of Education Key Lab of Environment and Health, School of Public Health, Tongji Medical College, Huazhong University of Science and Technology, Wuhan, China; 4 Department of Gastroenterology, Shenzhen Clinical Medical College, Guangzhou University of Traditional Chinese Medicine, Shenzhen, China; 5 Department of Allergy, Tongji Hospital of Tongji Medical College, Huazhong University of Science and Technology, Wuhan, China

## Abstract

Several studies have reported a positive association between caesarean section for expeditious pregnancy termination and perinatal outcomes in acute fatty liver of pregnancy (AFLP); however, the risks remain unclear and independent studies have reported conflicting findings. In this meta-analysis, we aimed to confirm the relationship between caesarean section and perinatal outcomes in AFLP. The PubMed, Embase, and China National Knowledge Infrastructure databases were searched (until July 17, 2015) for observational clinical studies focusing on the association between caesarean section and perinatal outcomes in AFLP. Data were extracted and processed independently by 2 authors. We also compared caesarean section with vaginal delivery to further investigate this relationship. We observed that 2 of the 3 primary outcomes in caesarean section exhibited positive effects—the maternal mortality rate was 44% lower (relative risk [RR], 0.56 [0.41–0.76]) and perinatal mortality rate was also reduced (RR, 0.52 [0.38–0.71]), compared to those for vaginal delivery. We did not find any associations between caesarean section and perinatal outcomes in AFLP in terms of neonatal mortality type and maternal multiple organ complications. These findings emphasise the significant prognostic value and clinical implications of caesarean section in AFLP, and suggest that the adverse outcomes should be reduced.

Acute fatty liver of pregnancy (AFLP)—first described as a specific clinical entity in 1940 by Sheehan—is a rare but potentially fatal condition of the third trimester of pregnancy^1^. AFLP is characterised by profound liver failure and may be accompanied by renal failure, disseminated intravascular coagulation (DIC), hypoglycaemia, and encephalopathy^2^. The incidence of this condition can vary widely, from 1 in 900 to 1 in 16,000 deliveries^3^,^4^. Although the maternal and foetal mortality rates in cases of AFLP have reportedly reached up to 80% in the past few decades^2^, these values have been gradually declining as a result of early diagnosis of the disease and prompt termination of pregnancy^5^,^6^,^7^,^8^.

The involvement of a multidisciplinary team in a critical care environment may play a crucial role in ensuring that patients with AFLP receive supportive treatment. Nevertheless, the diagnosis of AFLP remains challenging. In cases of AFLP where the condition is identified, the termination of pregnancy is the only treatment available to achieve the most desirable outcomes for both the mother and foetus. However, the guidelines for selecting the delivery mode among women with AFLP are lacking. Studies on the ideal delivery mode, elective caesarean section, or trial of vaginal delivery have yielded inconsistent findings^5^. Some researchers found that caesarean delivery is hazardous for the mother, and instead recommend labour induction with close monitoring, since the latter decreases the morbidity of maternal complications^9^,^10^. In contrast, some other authors recommend the use of caesarean section to improve the foetal prognosis, even though it increases the risk of maternal complications^9^,^11^,^12^. Patients with AFLP, who also present with coagulopathy, usually have a high risk of maternal haemorrhage complications when undergoing caesarean section^5^,^6^,^13^,^14^,^15^,^16^. Hence, the delivery approach should be carefully selected based on the condition of the mother and foetus. Given the potential for adverse outcomes, the measures for prenatal and postpartum care of women with AFLP have been carefully tailored according to existing guidelines^17^. Nevertheless, the current evidence on the ideal delivery mode to be adopted in clinical practice is limited, and additional evidence is required^12^. At present, most doctors prefer to choose caesarean selection for AFLP patients based on their clinical experience rather than a further assessment of the risks^18^,^19^.

In the present study, we performed a systematic, comprehensive meta-analysis of the available evidence to determine the maternal and perinatal outcomes in AFLP between caesarean section and vaginal delivery. Considering the serious adverse prognosis of AFLP and the importance of the delivery mode in AFLP, researchers and administrators need a method to quantify the extent of risk of the delivery mode in adverse pregnancy outcomes.

## Methods

### Search strategy

A systematic literature search of the following databases was conducted from inception until July 17, 2015: Embase, PubMed, and China National Knowledge Infrastructure. Bibliographies from the retrieved primary articles were also reviewed via manual searching. Our search terms included: “acute fatty liver of pregnancy,” “acute fatty liver in pregnancy,” “pregnancy-related liver diseases,” “obesity and acute fatty liver of pregnancy,” “fatty liver and pregnancy,” and “abnormal metabolism and acute fatty liver of pregnancy.”

### Study eligibility criteria

#### Inclusion criteria

The study sample comprised case-control studies, cohort studies, and cross-sectional studies that reported the clinical outcomes or complications of AFLP patients as the outcome(s) of interest, wherein the full text was published in Chinese or English. The study population was restricted to AFLP patients with a clear diagnosis. Studies were included if the mode of delivery was described in the text or tables. We observed that old age, primipara status, obesity, male sex, multiple pregnancy, and gene mutation in pregnant woman were underlying risk factors for peripartum maternal and foetal mortality in AFLP. Since AFLP is a rare condition, with a low incidence but high mortality, it was not feasible to analyse the data hierarchically by risk factors in the present study.

#### Exclusion criteria

Because the assessment of study quality/bias may be inadequate in studies that required translation, the studies for which the full text was not available in Chinese or English were excluded. Furthermore, studies involving less than 5 participants; non-human studies; and studies published only as case reports, abstracts, conference proceedings, reviews, and editorials were excluded. Moreover, studies without any relevant raw or adjusted data or those without outcomes of interest were excluded. If the publications appeared to overlap, in terms of the institution and study subjects, the overlapping portions of the studies were excluded. If patients with AFLP were also diagnosed with pregnancy-related liver disease or other liver diseases, or if they chose abortion in the studies, those studies were excluded.

### Outcome measures

#### Primary outcomes

Our primary outcomes included maternal mortality, and perinatal and neonatal mortality.

#### Secondary outcomes

We also studied a number of secondary maternal outcomes, including liver failure-associated complications (hypoglycaemia, DIC, ascites, and encephalopathy), other organ injuries (renal insufficiency, pancreatitis, and multiple organ dysfunction syndrome [MODS]), obstetric haemorrhage, and infection.

### Study selection

Two evaluators (Q.J. and H.-Y.W.) independently reviewed the titles and abstracts of all the searched citations. If either reviewer considered that the citation had a potential relationship and a low threshold, the full-text was retrieved and independently assessed by the 2 reviewers. Disagreements were resolved through discussion and consensus with a third senior investigator (H.S.).

### Data extraction

Two investigators (Q.J. and H.-Y.W.) checked the study eligibility and independently extracted the following data from each study: publication year, first author’s surname, study origin (city, state, country), study design and period, interval between first symptom and delivery, basic maternal information (weeks of pregnancy, age, parity, and multiple gestation), route of delivery, maternal death, foetal death, foetal sex, maternal complications (ascites, renal dysfunction/renal failure, coagulopathy/DIC, hypoglycaemia, encephalopathy, secondary infection, pancreatitis, obstetrics-related outcomes, and MODS), length of hospital stay, study inclusion and exclusion criteria, association between delivery route and maternal and foetal outcomes, and information on bias/study quality. We recorded the available data from the publications via the consensus process described earlier.

### Data synthesis

The available data were analysed via a formal meta-analysis. Forest plots were used to exhibit the effect estimates of individual and aggregate parameters and their corresponding confidence intervals (CI). Relative risk (RR) with 95% CI was selected as an effect measure for all outcomes^20^. The studies in the meta-analysis were weighted according to the inverse variance of the study. Heterogeneity between the studies was evaluated and reported by tau-square tests and Q tests for significance; P < 0.05 was considered statistically significant. Statistical inconsistency between the studies was measured and quantified using I^2^ tests^21^. If heterogeneity was significant between the studies (P < 0.05 or I^2^ > 50%), a fixed-effects model was not adopted, but instead, a random-effects model was applied. Sensitivity analysis was performed to evaluate whether the results could have been obviously affected by a single study. Publication bias was assessed with the use of contour-enhanced funnel plots, and bias was considered to be present when the P value was less than 0.10. Furthermore, the primary outcomes between caesarean section and vaginal delivery were also assessed in subgroups based on race: Asian vs Caucasian.

### Quality assessment

Study quality was assessed according to a modified version of the Newcastle-Ottawa Scale^22^. Due to the lack of universally accepted cut-offs for high vs low quality studies, subgroup analyses based on study quality were not planned. However, the results of forest plots yielded related information of study quality to ensure simultaneous assessment.

## Results

### Study characteristics

**Figure 1:**
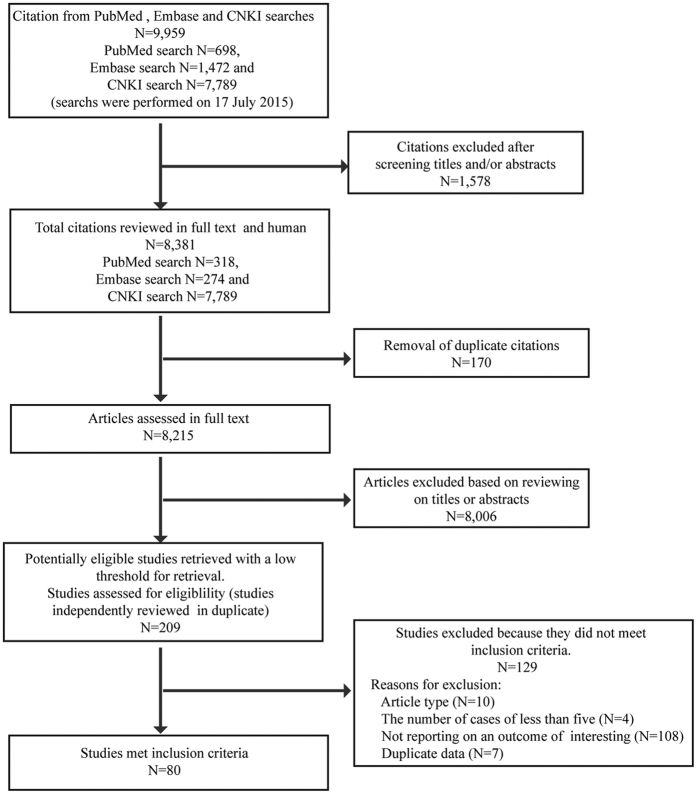
Study process of systematic review and meta-analysis of effect of caesarean section on maternal and foetal outcomes in AFLP.

A total of 8,381 full-text citations, limited to the human species, were identified in our search (Fig. 1). After excluding duplicate citations and reviewing the titles or abstracts, 209 full-text articles were identified. The most common reasons for exclusion included the lack of reporting on an outcome of interest, duplicate data, and article type (e.g., reviews, conference proceedings, and case reports). Of these, 80 studies—including 78 cohort studies and 2 case-control studies—met the inclusion criteria for this review. Study quality was assessed according to a modified version of the Newcastle-Ottawa Scale. The average quality score of the 78 cohort studies was 8.4 (range, 5–9) and the quality score of the 2 case-control studies was 2; further detail is provided in Supplementary Tables S1 and S2.

With regard to general information of the 1,350 subjects from the selected studies, the average patient age was 25.2 years (range, 15–42 years) and the mean gestational age was 36 weeks (range, 26–42 weeks). The mean interval from occurrence of AFLP to delivery was 8.14 days (range, 0.5–40 days). Moreover, a total of 84.63% (1,085/1,282) of patients were primigravida and 15.37% (197/1,282) of patients were multigravida. Among the 1,211 subjects, there were 876 cases of single and 335 cases of multiple pregnancy. Furthermore, foetal sex was male in 443 cases (69.44%) and female in the other 195 cases (30.56%).

### Maternal mortality, and perinatal and neonatal mortality

**Figure 2:**
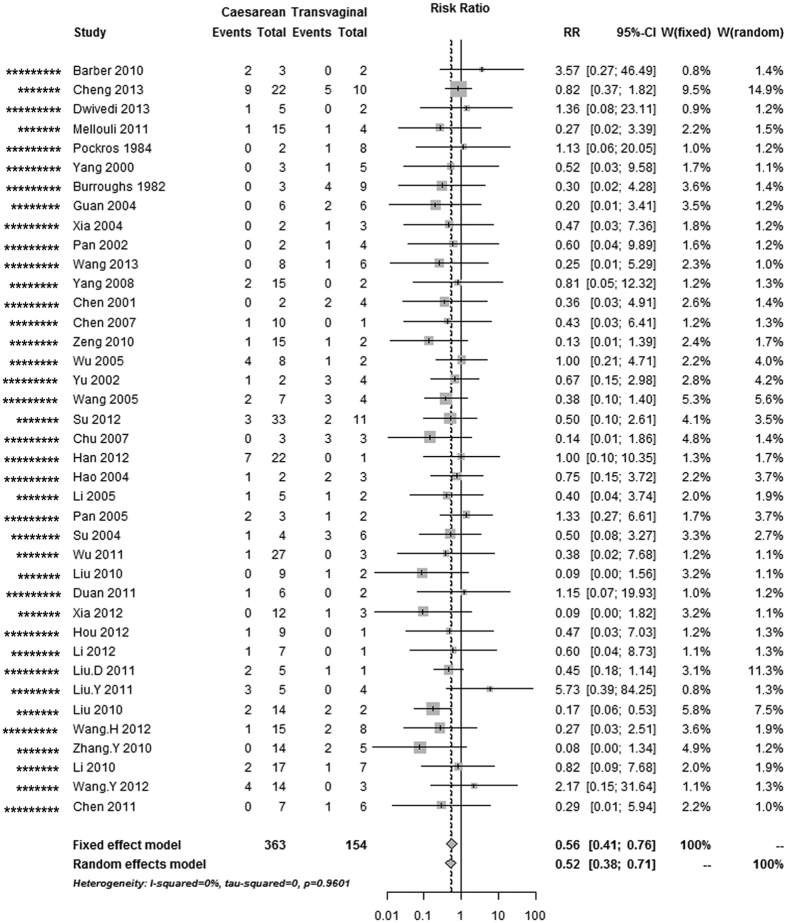
Forest plot of the unadjusted risk of maternal death in AFLP patients with caesarean section compared with vaginal delivery from cohort studies. ^*^Number of stars showing study quality based on the Newcastle–Ottawa Scale rating (possible maximum 9 stars).

**Figure 3:**
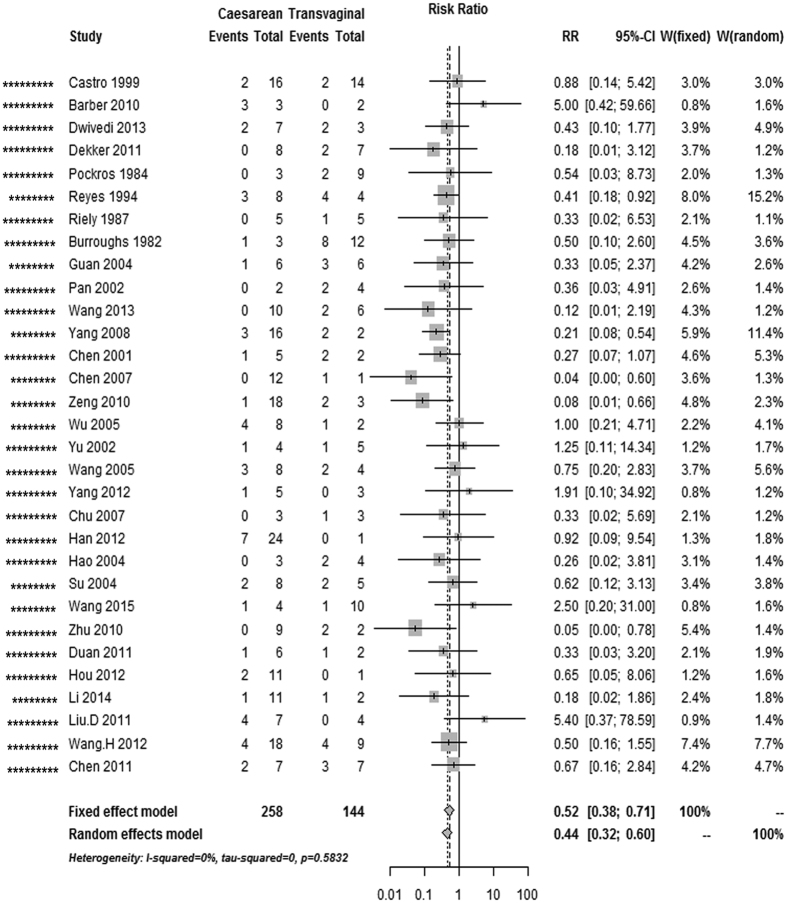
Forest plot of the unadjusted risk of perinatal death in AFLP patients with caesarean section compared with vaginal delivery from cohort studies. ^*^Number of stars showing study quality based on the Newcastle–Ottawa Scale rating (possible maximum 9 stars).

**Figure 4:**
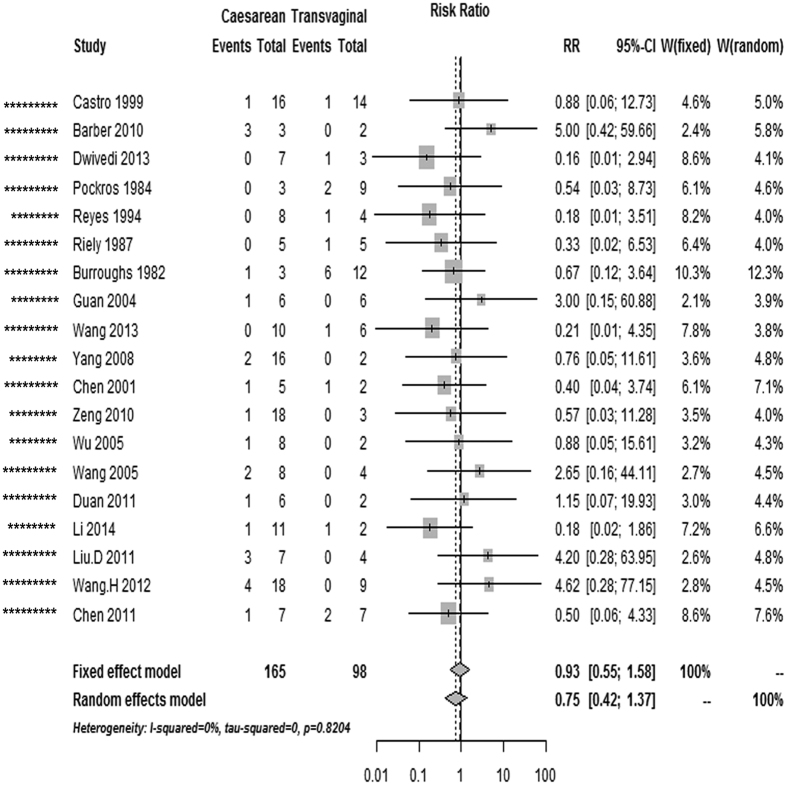
Forest plot of the unadjusted risk of neonatal death in AFLP patients with caesarean section compared with vaginal delivery from cohort studies. ^*^Number of stars showing study quality based on the Newcastle–Ottawa Scale rating (possible maximum 9 stars).

The overall risk of maternal mortality in AFLP patients was 44% lower in cases with caesarean section than in cases with vaginal delivery (pooled RR, 0.56 [0.41–0.76], 39 studies; Table 1, Fig. 2); this decrease in the perinatal mortality risk remained significant in the caesarean section and vaginal delivery categories (pooled RR, 0.52 [0.38–0.71], 31 studies; Fig. 3). Nevertheless, the risk of neonatal mortality differed between caesarean section and vaginal delivery (pooled RR, 0.93 [0.55–1.58], 19 studies; Fig. 4). As there was no evidence of significant heterogeneity between the above-mentioned studies (I^2^ < 50%, P > 0.05), fixed-effects models were adopted.

When the data on primary outcomes were assessed according to the ethnic background, the major difference in sample size, in particular the small number of Caucasian patients, limited our power to draw any solid conclusions about the impact of race. Results are shown in the Table 1.

### Maternal complications

Most studies found that women with a definitive diagnosis of AFLP accompanied by a series of postpartum complications such as liver failure, renal insufficiency, DIC, ascites, encephalopathy, postpartum haemorrhage, pancreatitis, infection, and MODS, usually exhibited serious maternal morbidity and mortality rates. Based on the current literature, the overall incidence of relevant maternal clinical complications associated with AFLP is summarised in Table 2.

There were no significant differences between the women with AFLP who underwent caesarean section and those with AFLP who underwent vaginal delivery in terms of liver failure-associated complications (hypoglycaemia, DIC, ascites, and encephalopathy), other organ injuries (renal insufficiency, pancreatitis, and MODS), obstetric haemorrhage, and infection (Table 3).

### Sensitivity analyses

A series of sensitivity analyses were conducted by omitting a single study and calculating the pooled RR for the remaining studies under the fixed-effects model, in order to assess the effects of each individual study on the pooled RR. The results of sensitivity analysis are shown in Supplementary Figs S1–S3. None of the interventions could affect the final results of the meta-analysis. Thus, the sensitivity analyses indicated that the pooled RR between caesarean section and maternal, perinatal, and neonatal mortality were reliable and stable.

### Publication bias

There was no evidence of any publication bias based on a visual inspection of the funnel plots. The funnel plot for the association between caesarean section and maternal mortality did not show the asymmetry that is typically associated with publication bias; in fact, the P value for Egger’s regression asymmetry test was 0.61, which indicated a low probability of publication bias (Supplementary Fig. S4). The trends were nearly identical between caesarean section and perinatal and neonatal mortality (P = 0.61 and P = 0.72, Supplementary Figs S5 and S6).

## Discussion

Although peripartum maternal and foetal mortality in AFLP is known to be associated with primipara, multipara, male foetus, and defects in genes concerning fatty acid oxidation^23^,^24^,^25^,^26^,^27^, one intriguing finding was the choice of the delivery mode. As with any relatively rare disease, it is not feasible to make firm recommendations for AFLP based on the currently limited literature. AFLP has never been resolved by methods other than expeditious delivery, although it is still unclear whether delivery should involve caesarean section or labour induction^5^,^12^,^28^. Given the probably lethal condition of late pregnancy in AFLP, any potential causal association between peripartum mortality in AFLP and the mode of pregnancy termination could have a profound effect on public health outcome.

To our knowledge, this is the first comprehensive systematic review and meta-analysis on this topic. We observed that women with AFLP in late pregnancy who underwent caesarean section delivery had a significantly lower risk of peripartum mortality, compared to those who underwent vaginal delivery. Moreover, several neonatal and other maternal life-threatening complications such as liver failure, DIC, and MODS were found to be relatively rare in AFLP patients who underwent caesarean section delivery, although there was no significant difference in the pooled RR between caesarean section and vaginal delivery in this small hospital-based case series. Another possible explanation for this lack of statistical difference between caesarean section and vaginal delivery may involve the large heterogeneity of the clinical-based data, including a deviation of the diagnosis and/or supportive care depending on the overall disease severity.

Our subgroup analyses enabled us to explore the potential background and the cause of the decrease in adverse outcomes. With maternal mortality, for instance, we noted that the reduction in the risk is multifactorial, both from basic maternal factors as well as medical factors. The reduction in the risk may be associated with the maternal physical features since the differences in body size and pelvic structure will give rise to the different obstetrical indications and results of delivery mode in patients with AFLP. It may also be related to the severity of AFLP, as caesarean section may be more appropriate to terminate pregnancy in more severe cases. On the other hand, medical factors, such as a longer interval prior to delivery, may contribute to increase the mortality risk of women with AFLP^12^,^29^. Moreover, previous investigators have emphasised that the current clinical practice worldwide favours caesarean section delivery^7^,^8^,^16^,^30^, and in most women, particularly Asians, delivery is primarily conducted electively before labour onset^12^,^18^.

The salient features of this systematic review and meta-analysis include the extensive assessment of the ante/postpartum outcomes in AFLP in the mother and the foetus, which facilitated a general evaluation of the risks of caesarean section. We conducted a series of sensitivity analyses that helped detect heterogeneity, discover significant associations, and explore underlying differences between the population subgroups. In addition, we performed a well-thought-out quality assessment of each included study and integrated it with the overall results; accordingly, it was reflected in the forest plots as a combined evaluation of the quality of the study with the degree of risk it reported.

The present study had certain limitations. First, case-control studies and cohort studies may be susceptible to detection bias, and selection and recall biases are also common in retrospective or case-control studies. In the present literature review, these biases are indeed unavoidable. One important reason for such biases may be that the delivery route could be influenced by the condition of the patient (worse or better) in terms of severity and outcomes. For example, the patient may be too ill to undergo a major surgery due to the high risk of fatal complications, such as haemorrhagic shock, DIC, or accidents during anaesthesia administration, or the patient may be too ill to tolerate a time-consuming delivery that could cause rapid progression of organ failure. However, the effect of confounding variables is negligible as the constituent ratio of patient’s condition is similar in the two delivery categories. In addition, the bias by route of delivery by indications does not negate the importance of our findings since delivery, regardless of delivery route, will not completely eliminate the risk of adverse outcomes in AFLP. Second, as the studies included in this meta-analysis were all observational studies, the observed positive association between the mode of delivery and maternal and foetal outcomes may have resulted from other unmeasured factors. In particular, the maternal and foetal mortality rates due to vaginal delivery may have been higher due to the poor quality of antenatal care, misdiagnosis, or inappropriate treatment (lack of treatment or delayed or incorrect treatment) of AFLP in certain studies. Furthermore, the sample size of cases that received vaginal delivery in the present study was small, as many such cases had incomplete data about the disease in the early stages. Third, most of the studies selected in our meta-analysis lacked information on detailed history taking and the interval from occurrence of AFLP to delivery. Hence, we were unable to develop a severity-dependent analysis to more precisely assess the relationship between these variables. Furthermore, the data were not of sufficient quality to ensure appropriate foetal complication analyses, including respiratory distress syndrome, assisted ventilation, neonatal intensive care unit/special care unit admission, and hospital stay, thus limiting our power to draw any solid conclusions.

Despite these limitations, this systematic review and meta-analysis thoroughly evaluated the association between the mode of pregnancy termination and the perinatal mortality in AFLP. We observed that caesarean section is associated with better pregnancy outcomes, and based on current evidence, caesarean section is the safest method of delivery and should be recommended to lower the risk of adverse pregnancy outcomes in AFLP. Nevertheless, additional optimally designed studies that consider the possible confounding factors could eventually provide a better, comprehensive understanding of the association between perinatal mortality and the mode of pregnancy termination in AFLP.

## Additional Information

**How to cite this article**: Wang, H.-Y. *et al*. Effect of caesarean section on maternal and foetal outcomes in acute fatty liver of pregnancy: a systematic review and meta-analysis. *Sci. Rep.* **6**, 28826; doi: 10.1038/srep28826 (2016).

## Supplementary Material

Supplementary Information

## Figures and Tables

**Table 1:** Summary of primary outcomes in studies of caesarean section compared with vaginal delivery in AFLP, unadjusted results.

Outcomes	Caesarean section vs Vaginal delivery				
	Total N	Total N	Pooled unadjusted*	I^2^ (%)	P#
	studies	participants	RR (95% CI)		
Maternal death	39	517	**0**.**56** (**0**.**41**–**0**.**76**)	0.00	0.96
White/Caucasian	4	46	0.74 (0.23–2.40)	0.00	0.46
Asian	35	471	0.54 (0.39–0.75)	0.00	0.96
Perinatal death	31	402	**0**.**52** (**0**.**38**–**0**.**71**)	0.00	0.58
White/Caucasian	7	99	0.62 (0.33–1.17)	0.00	0.57
Asian	24	303	0.49 (0.34–0.70)	0.00	0.48
Neonatal death	19	263	0.93 (0.55–1.58)	0.00	0.82
White/Caucasian	6	84	0.80 (0.33–1.95)	0.00	0.62
Asian	13	179	1.01 (0.52–1.95)	0.00	0.70

Bolding indicates statistically significant results. ^*^Fixed effect model; ^#^The Q test was employed to evaluate the heterogeneity.

**Table 2:** Summary of incidence of maternal complications in AFLP, unadjusted results.

Complications	Total N	Total N	Pooled unadjusted*	I^2^ (%)	P#
	studies	participants	incidence (95% CI)		
Liver failure	22	309	0.42 (0.26–0.58)	94.40	0.00
Hypoglycemia	28	449	0.57 (0.44–0.70)	94.30	0.00
Ascites	15	205	0.45 (0.26–0.64)	91.80	0.00
DIC	42	686	0.39 (0.30–0.49)	88.90	0.00
Encephalopathy	46	730	0.37 (0.30–0.44)	78.40	0.00
Renal insufficiency	27	503	0.66 (0.56–0.77)	93.80	0.00
Pancreatitis	14	279	0.11 (0.05–0.17)	63.20	0.00
MODS	36	642	0.67 (0.56–0.78)	95.30	0.00
Apparent infection	23	335	0.29 (0.21–0.38)	74.60	0.00

Bolding indicates statistically significant results. ^*^Random effect model; ^#^The Q test was employed to evaluate the heterogeneity.

**Table 3:** Summary of secondary maternal outcomes in studies of caesarean section compared with vaginal delivery in AFLP, unadjusted results.

Complications	Caesarean section vs Vaginal delivery				
	Total N	Total N	Pooled unadjusted	I^2^ (%)	P#
	studies	participants	RR (95% CI)		
Liver failure	11	100	0.87 (0.61–1.24)	0.00	0.81
Hypoglycemia	6	62	0.93 (0.62–1.39)	0.00	0.46
Ascites	8	62	1.50 (0.91–2.49)	31.50	0.21
DIC	24	220	0.85 (0.65–1.11)	0.00	0.68
Encephalopathy	18	174	0.80 (0.51–1.27)*	55.20	0.01
Renal insufficiency	20	174	1.10 (0.85–1.44)	0.00	0.83
Pancreatitis	5	46	1.46 (0.39–5.43)	25.90	0.26
MODS	15	138	0.86 (0.69–1.07)	19.30	0.25
Apparent infection	12	116	1.16 (0.66–2.04)	20.00	0.25
Postpartum haemorrhage	27	315	0.92 (0.71–1.18)	0.00	0.76

Bolding indicates statistically significant results. ^*^Random effect model; ^#^The Q test was employed to evaluate the heterogeneity.
