# Supplementary material for: Effect of caesarean section on maternal and foetal outcomes in acute fatty liver of pregnancy: a systematic review and meta-analysis
Source: Sci Rep. 2016 Jul 8;6:28826. doi: 10.1038/srep28826 (PMC4937371; doi:10.1038/srep28826)
Supplement: Supplementary Information [file srep28826-s1.pdf]

Effect of caesarean section on maternal and foetal outcomes in acute fatty liver of pregnancy: a systematic review and meta-analysis

Hong-Yan Wang[1,4,#], Qing Jiang[2,5,#], Hao Shi[3], Yun-Qing Xu[2], Ai-Chao Shi[1], Yuan-Li Sun[2], Jian Li[4], Qin Ning[1,*] & Guan-Xin Shen[2,*]

[1]Department of Infectious Disease, Tongji Hospital of Tongji Medical College, Huazhong University of Science and Technology, Wuhan, China; [2]Department of Immunology, School of Basic Medicine, Tongji Medical College, Huazhong University of Science and Technology, Wuhan, China; [3]Department of Epidemiology and Biostatistics, and the Ministry of Education Key Lab of Environment and Health, School of Public Health, Tongji Medical College, Huazhong University of Science and Technology, Wuhan, China; [4]Department of Gastroenterology, Shenzhen Clinical Medical College, Guangzhou University of Traditional Chinese Medicine, Shenzhen, China; [5]Department of Allergy, Tongji Hospital of Tongji Medical College, Huazhong University of Science and Technology, Wuhan, China.

[#]Contributed equally.

[*]Corresponding authors at: Department of Infectious Disease, and Department of Immunology, Tongji Medical College, Huazhong University of Science and Technology, Wuhan 430030, China.

E-mail address: shenguanxin@hust.edu.cn(G.-X.S.); qning@vip.sina.com(Q.N.).

Table S1. Quality assessment cohort studies included in systematic review and meta-analysis of effect of caesarean section on maternal and foetal outcomes in AFLP.

**Study**	**Overall Quality Assessment**	**Selection**				**Comparability**	**Outcome**		
		**Representativeness of Exposed Cohort**<br><br>★= Representative of an average pregnant woman in a community<br><br>(no points allocated if >20% excluded due to missing information or if no description of derivation of cohort)	**Selection of Non-exposed Cohort**<br><br>★= Drawn from the same community as the exposed cohort<br><br>(no points allocated if drawn from a different source or if no description of derivation of cohort)	**Ascertainment of Exposure**<br><br>★= Measured<br><br>(no points allocated if self-reported or no description)	**Demonstration that outcome of interest was not present at start of study**<br>★= yes<br><br>(no points allocated if no description)	**Comparability of Cohorts of Design or Analysis**<br><br>★= Study controls for confounder(s) (up to 2 stars if ≥ 2 confounders addressed)<br>(no points allocated if study fails to control for confounders or no description)	**Assessment of Outcome**<br><br>★= Medical chart or record linkage<br><br>(no points allocated if self-reported or no description)	**Was follow-up long enough for outcomes to occur**<br>★= select an adequate follow up period for outcome of interest<br><br>(no points allocated if no description)	**Adequacy of Follow-up of Cohorts**<br><br>★= Follow up rate ≥90%, or if ≥80%, those lost reported to be similar to those followed<br><br>(no points allocated if follow up <90% with no description of those lost, or <80%, or no description)
Castro 1999	9/9	★	★	★	★	★★	★	★	★
Barber 2010	9/9	★	★	★	★	★★	★	★	★
Gracia 2011	7/9	★	★	★	★	★	★	★	
Cheng 2013	7/9	★	★	★	★	★	★	★	
Wei 2010	8/9	★	★	★	★	★	★	★	★
Dwived 2013	9/9	★	★	★	★	★★	★	★	★
Xiong 2015	7/9	★	★	★	★	★	★	★	
Mellouli	7/9	★	★	★	★	★	★	★	

2011									
Martin Jr. 2008	8/9	★	★	★	★	★	★	★	★
Mjahed 2006	7/9	★	★	★	★	★	★	★	
Dekker 2011	9/9	★	★	★	★	★★	★	★	★
Nelson 2013	5/9	★	★	★	★	★			
Fesenmeier 2005	7/9	★	★	★	★	★	★	★	
Lau 2010	5/9	★	★	★	★	★			
Pockros 1984	9/9	★	★	★	★	★★	★	★	★
Reyes 1994	8/9	★	★	★	★	★	★	★	★
Riely 1987	9/9	★	★	★	★	★★	★	★	★
Rolfes 1985	5/9	★	★	★	★	★			
Usta 1994	5/9	★	★	★	★	★			
Yang 2000	9/9	★	★	★	★	★★	★	★	★
Bahloula 2006	5/9	★	★	★	★	★			
Burroughs 1982	9/9	★	★	★	★	★★	★	★	★
Zhou 2013	6/9	★		★	★		★	★	★
Knight 2008	5/9	★	★	★	★	★			
Guan 2004	8/9	★	★	★	★	★	★	★	★
Xia 2004	9/9	★	★	★	★	★★	★	★	★
Pan 2002	9/9	★	★	★	★	★★	★	★	★
Wang 2013	9/9	★	★	★	★	★★	★	★	★
Yang 2008	8/9	★	★	★	★	★	★	★	★
Chen 2001	9/9	★	★	★	★	★★	★	★	★
Chen 2007	8/9	★	★	★	★	★	★	★	★
Zeng 2010	8/9	★	★	★	★	★	★	★	★

Chen 2014	8/9	★	★	★	★	★	★	★	★
Wu 2005	8/9	★	★	★	★	★	★	★	★
Yu 2002	9/9	★	★	★	★	★★	★	★	★
Guo 2001	8/9	★	★	★	★	★	★	★	★
Wang 2005	9/9	★	★	★	★	★★	★	★	★
Yang 2012	9/9	★	★	★	★	★★	★	★	★
Hao 2007	9/9	★	★	★	★	★★	★	★	★
Su 2012	7/9	★	★	★	★	★	★	★	
Chu 2007	9/9	★	★	★	★	★★	★	★	★
Han 2012	9/9	★	★	★	★	★★	★	★	★
Hao 2004	9/9	★	★	★	★	★★	★	★	★
Hao 2002	8/9	★	★	★	★	★	★	★	★
Li 2005	7/9	★	★	★	★	★	★	★	
Pan 2005	9/9	★	★	★	★	★★	★	★	★
Zhang 2010	9/9	★	★	★	★	★★	★	★	★
Yu 2000	9/9	★	★	★	★	★★	★	★	★
Su 2004	8/9	★	★	★	★	★	★	★	★
Wu 2011	7/9	★	★	★	★	★	★	★	
Lv 2007	7/9	★	★	★	★	★	★	★	
Ye 2004	8/9	★	★	★	★	★★	★	★	
Zeng 2013	5/9	★	★	★	★	★			
Wang 2015	8/9	★	★	★	★	★	★	★	★
Zhu 2010	9/9	★	★	★	★	★★	★	★	★
Liu 2010	7/9	★	★	★	★	★	★	★	
Duan 2011	9/9	★	★	★	★	★★	★	★	★
Xia 2012	7/9	★	★	★	★	★	★	★	
Hou 2012	9/9	★	★	★	★	★★	★	★	★
Wang 2012	5/9	★	★	★	★	★			
Song 2011	6/9	★		★	★		★	★	★

Li 2014	8/9	★	★	★	★	★	★	★	★
Ding 2010	6/9	★		★	★		★	★	★
Lin 2013	5/9	★	★	★	★	★			
Li 2012	8/9	★	★	★	★	★★	★	★	
Yang 2005	6/9	★	★	★	★	★★			
Liu D. 2011	9/9	★	★	★	★	★★	★	★	★
Liu Y. 2011	8/9	★	★	★	★	★	★	★	★
Tang 2013	5/9	★	★	★	★	★			
Zhao 2011	5/9	★	★	★	★	★			
Liu 2010	7/9	★	★	★	★	★	★	★	
Wang 2012	9/9	★	★	★	★	★★	★	★	★
Cao 2012	7/9	★	★	★	★	★	★	★	
Zhang 2010	7/9	★	★	★	★	★	★	★	
Li 2015	6/9	★		★	★		★	★	★
Li 2010	7/9	★	★	★	★	★	★	★	
Wang 2012	7/9	★	★	★	★	★	★	★	
Chen 2011	9/9	★	★	★	★	★★	★	★	★

Table S2. Quality assessment case-control studies included in systematic review and meta-analysis of effect of caesarean section on maternal and foetal outcomes in AFLP.

Study	Overall Quality Assessment	Selection				Comparability	Outcome		
		**Is the case definition adequate?**<br><br>★= Yes, with independent validation (i.e., medical chart or record linkage)<br><br>(no points allocated if self-reported or no description)	**Representativeness of the cases**<br><br>★= Consecutive cases or appropriate sample (e.g., random sample)<br><br>(no points allocated if potential for selection bias or no description)	**Selection of Controls**<br><br>★= Community controls (i.e., same community as cases)<br><br>(no points allocated if hospital controls or no description)	**Demonstration that outcome of interest was not present at start of study**<br><br>★= yes<br><br>(no points allocated if "No" or no description)	**Comparability of Cohorts of Design or Analysis**<br><br>★= Study controls for confounder(s) (up to 2 stars if ≥ 2 confounders addressed)<br><br>(no points allocated if study fails to control for confounders or no description)	**Ascertainment of exposure**<br><br>★= Measured<br><br>(no points allocated if self-reported or no description)	**Same method of ascertainment for cases and controls**<br><br>★= Yes<br><br>(no points allocated if "No" or no description)	**Non-Response rate**<br><br>★= same rate for both groups<br><br>(no points allocated if non respondents described or if rate different and no designation)
Pereira 1997	2/9	★	★						
Westbrook 2010	2/9	★	★						

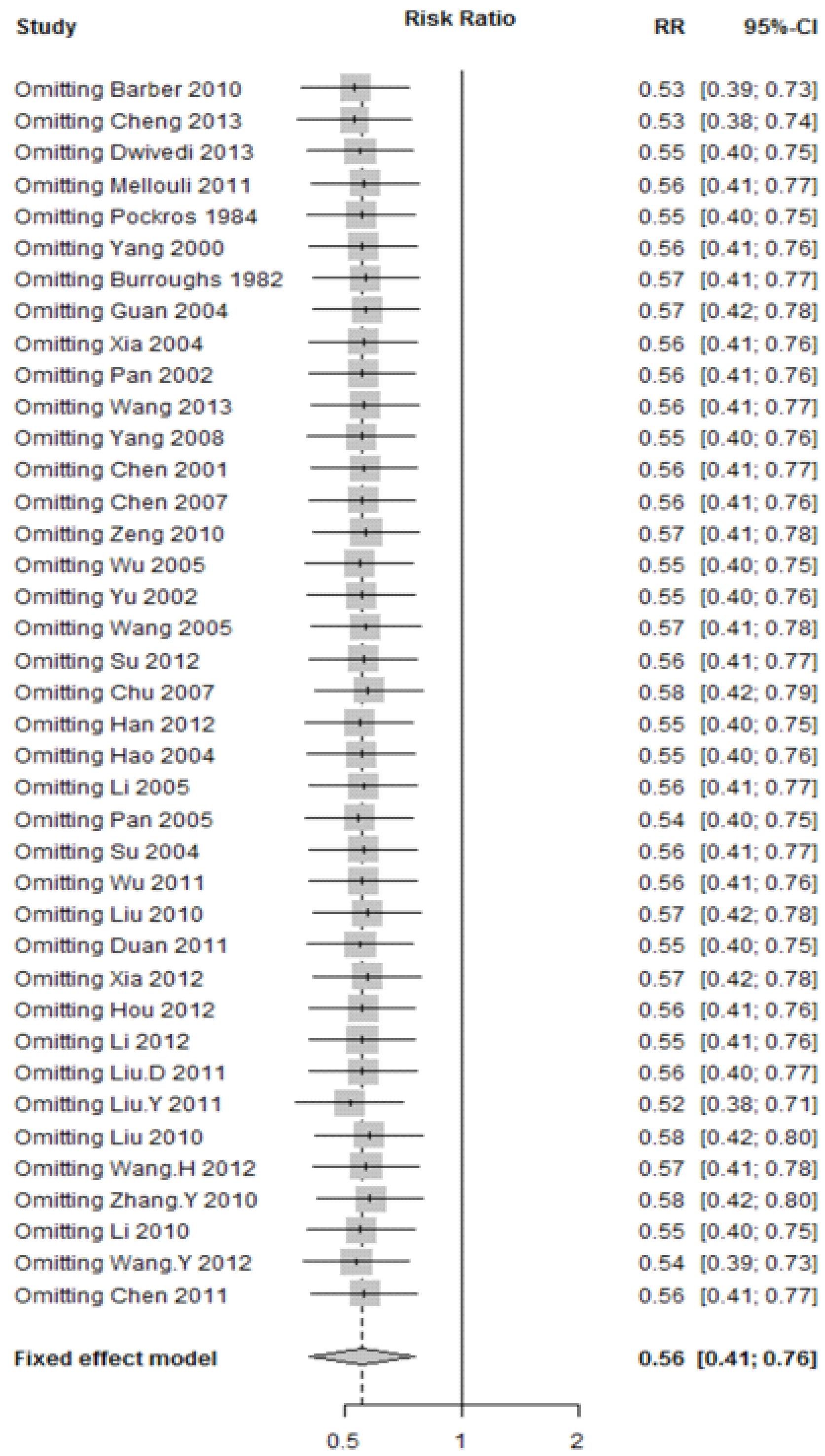

Figure S1. Sensitive plot of the unadjusted risk of maternal death in AFLP patients with caesarean section compared with vaginal delivery from cohort studies.

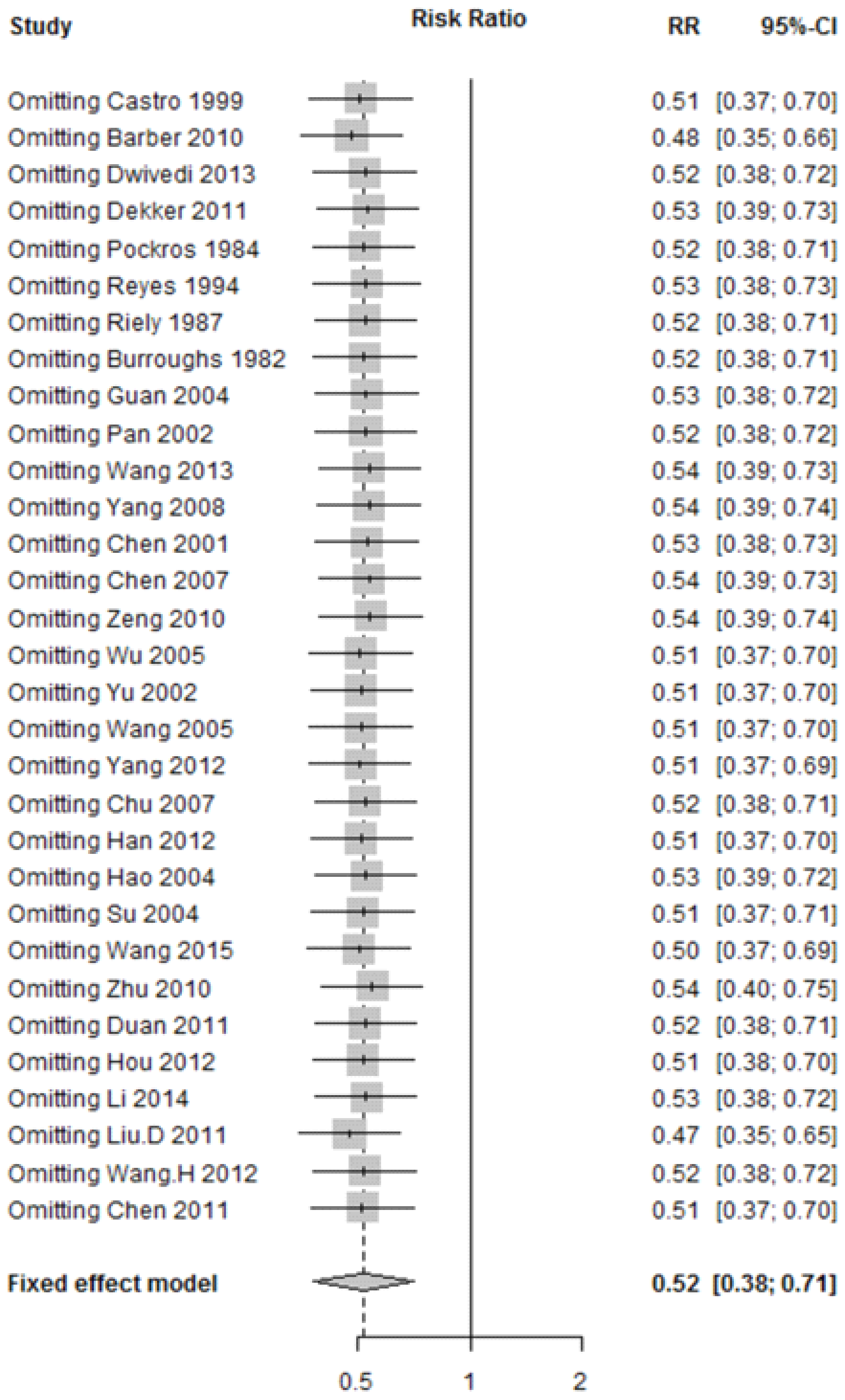

Figure S2. Sensitive plot of the unadjusted risk of perinatal death in AFLP patients with caesarean section compared with vaginal delivery from cohort studies.

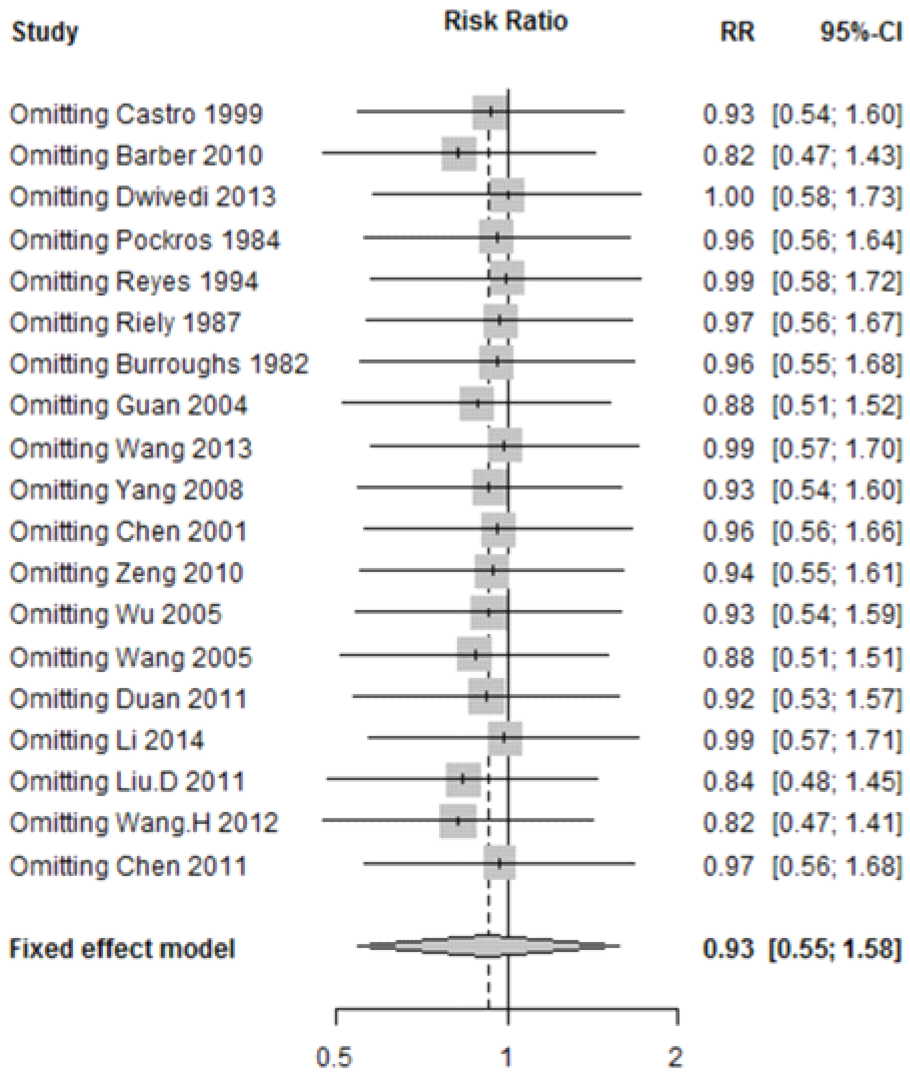

Figure S3. Sensitive plot of the unadjusted risk of neonatal death in AFLP patients with caesarean section compared with vaginal delivery from cohort studies.

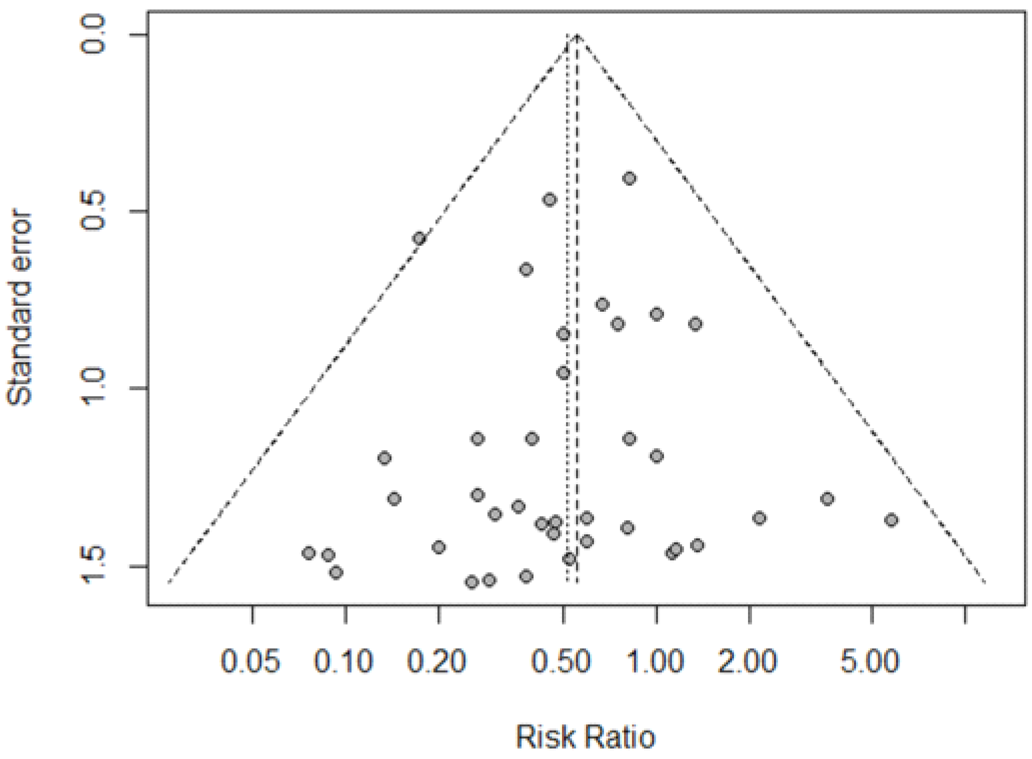

Figure S4. Funnel plot of the unadjusted risk of maternal death in AFLP patients with caesarean section compared with vaginal delivery from cohort studies.

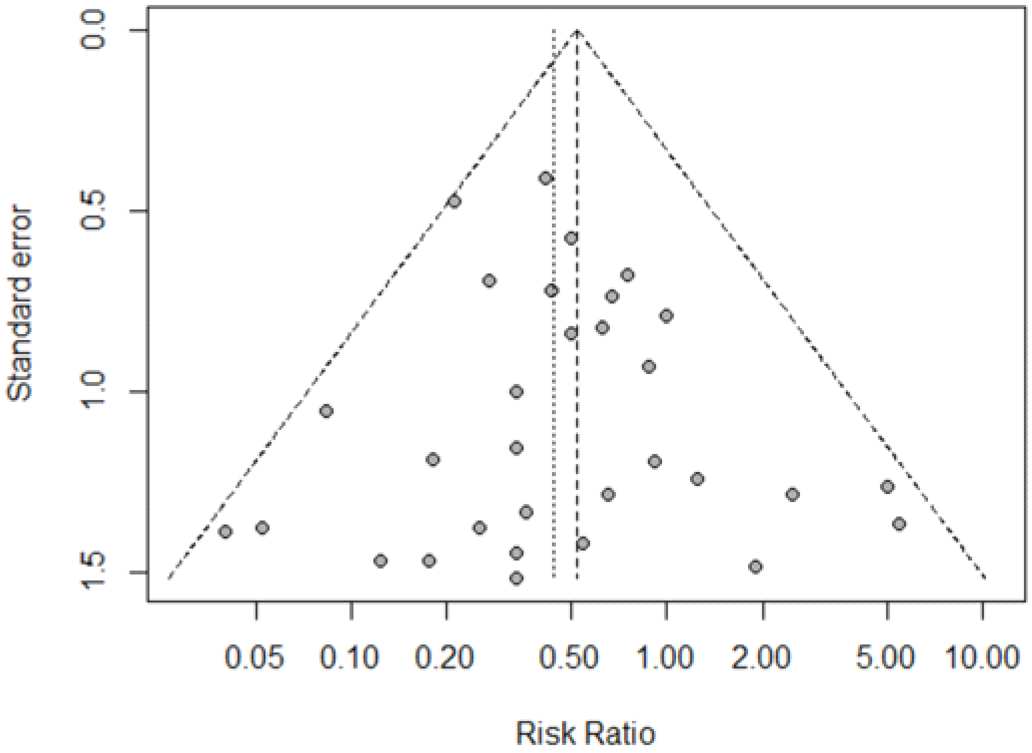

Figure S5. Funnel plot of the unadjusted risk of perinatal death in AFLP patients with caesarean section compared with vaginal delivery from cohort studies.

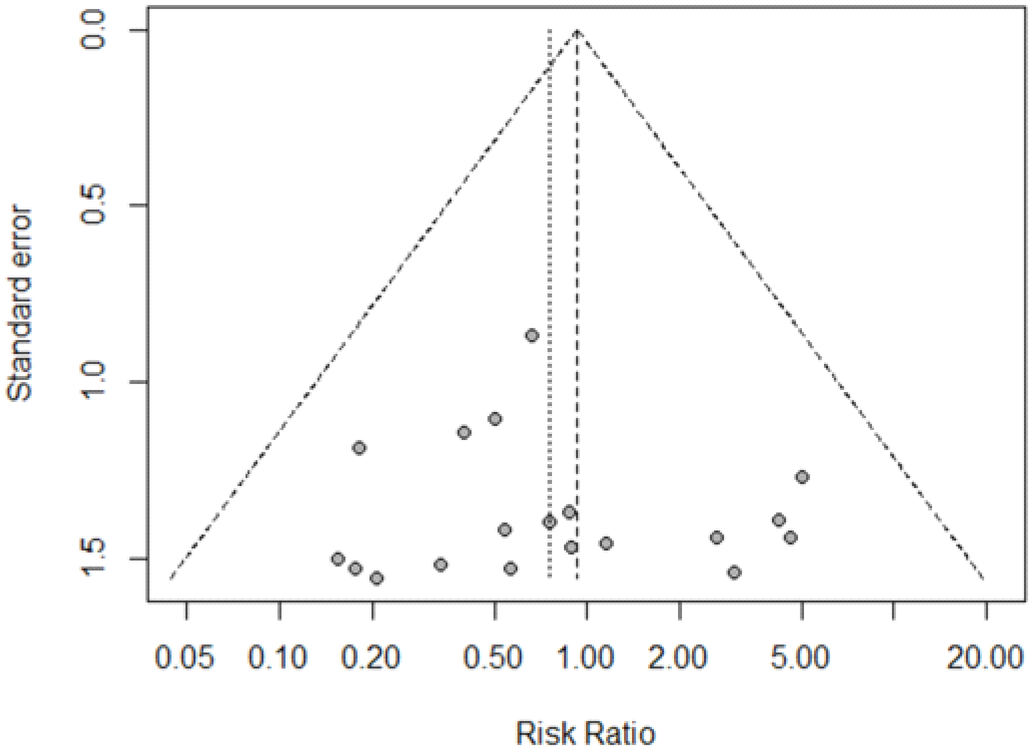

Figure S6. Funnel plot of the unadjusted risk of neonatal death in AFLP patients with caesarean section compared with vaginal delivery from cohort studies.
